# A proficient approach to forecast COVID-19 spread via optimized dynamic machine learning models

**DOI:** 10.1038/s41598-022-06218-3

**Published:** 2022-02-14

**Authors:** Yasminah Alali, Fouzi Harrou, Ying Sun

**Affiliations:** Computer, Electrical and Mathematical Sciences and Engineering (CEMSE) Division, King Abdullah University of Science and Technology (KAUST), Thuwal, 23955-6900 Saudi Arabia

**Keywords:** Health care, Mathematics and computing

## Abstract

This study aims to develop an assumption-free data-driven model to accurately forecast COVID-19 spread. Towards this end, we firstly employed Bayesian optimization to tune the Gaussian process regression (GPR) hyperparameters to develop an efficient GPR-based model for forecasting the recovered and confirmed COVID-19 cases in two highly impacted countries, India and Brazil. However, machine learning models do not consider the time dependency in the COVID-19 data series. Here, dynamic information has been taken into account to alleviate this limitation by introducing lagged measurements in constructing the investigated machine learning models. Additionally, we assessed the contribution of the incorporated features to the COVID-19 prediction using the Random Forest algorithm. Results reveal that significant improvement can be obtained using the proposed dynamic machine learning models. In addition, the results highlighted the superior performance of the dynamic GPR compared to the other models (i.e., Support vector regression, Boosted trees, Bagged trees, Decision tree, Random Forest, and XGBoost) by achieving an averaged mean absolute percentage error of around 0.1%. Finally, we provided the confidence level of the predicted results based on the dynamic GPR model and showed that the predictions are within the 95% confidence interval. This study presents a promising shallow and simple approach for predicting COVID-19 spread.

## Introduction

In December 2019, the world was waiting to welcome 2020; Wuhan hospital note unusual Severe Acute Respiratory by a new virus, and it was spread swiftly. They identify it later SARS-CoV-2 because of its similarity to the previous SARS CoV in 2002^1^. Sooner World Health Organization (WHO) calls this virus a novel coronavirus (nCOV-19) known as COVID-19. This virus can stay in the person for around 14 days without showing any symptoms that lead to transforms from the local epidemic of Wuhan to the global pandemic of the whole world. Because the early forecasting of the number of COVID-19 cases will help to control the incubation and non-spreading of the virus, the researchers and governments depend on machine learning (ML) which is part of artificial intelligence (AI) that can learn from the previous data to decide a solution in the real-world problem. In the COVID-19 pandemic problem, ML can predict the outbreak of COVID-19 for evaluating the riskiness of the virus and therefore raising the level of the procedures applied. The fact, the spread of the virus has receded in many countries when they use ML to detect COVID-19^2^.

In recent years, the effectiveness and benefit of the application of Artificial Intelligence (AI) have been proved in numerous sectors, such as healthcare, where it showed good performance as a decision support system to help identify diseases and make medical diagnoses^3–6^. During this pandemic, AI showed to be useful in predicting outbreaks and aid assemble quickly evolving data to support general health specialists in complex decision-making^7^. In addition, various AI-based tools were designed in the healthcare field^3,6,8^. For instance, a team at Boston Children’s Hospital developed an automated electronic information system called Health Map^9^. Notably, the Health Map employs real-time surveillance of emerging public health threats and unofficial online sources for observing disease outbreaks. Another example of an AI-based company specializing in infectious disease epidemiology is Blue Dot, which has flagged an alarm to its clients regarding the COVID-19 outbreak on December 31^3^. In addition, this company offered suitable predictions achievement for Zika virus in Brazil^10^. Also, we can find Google Flu, which employed search engine queries for enhancing the flu epidemic track. In^11^, authors introduced an intelligent framework for the COVID-19 telemedicine diagnostic via extended reality technology and deep learning networks. Specifically, an innovative Auxiliary Classifier Generative Adversarial Networks (ACGAN) is designed for COVID-19 prediction. This intelligent-based strategy can be viewed as a promising tool for supporting COVID-19 therapy and remote surgical plan cues. More improvement can be obtained by enhancing hardware design and deep learning models used in this Internet of Medical Things (IoMT) system. The authors in^12^ introduced a deep learning-driven approach for semi-supervised few-shot segmentation (FSS) of COVID-19 infection via radiographic images. The challenge addressed in this study is designing an effective and accurate segmentation of 2019-nCov infection based on small-sized annotated lung computed tomography (CT) scans. Essentially, the model was built semi-supervised using unlabeled CT slices and labeling one during training. Results based on publicly available COVID-19 CT scans revealed the superior performance of the FSS-2019-nCov compared to conventional models. However, the segmentation performance has not been tested on a large dataset. In^13^, a combined CNN-LSTM deep learning approach is introduced to detect COVID-19 cases based on X-ray images. More specifically, CNN has been employed as a feature extractor, and the LSTM is applied to CNN’s features to discriminate healthy people from the contaminated patients with COVID-19. They concluded that this approach outperformed the competitive CNN architectures by reaching an accuracy of 99.4%. However, the performance of this approach has been tested only on a relatively small-sized dataset; the generalizability of this approach needs to be verified using a large dataset. In addition, this approach cannot efficiently discriminate COVID-19 images containing different disease symptoms. Recently, the study in^14^ suggested an unsupervised detector combing a Variational Autoencoder(VAE) model with one-class SVM (1SVM) to detect COVID-19 infection using blood tests and reported accuracy of around 99%. Here, the VAE is used as a features extractor, and the 1SVM discriminates healthy patients from contaminated ones. Results showed the superior detection accuracy of this approach compared to Generative adversarial networks (GAN), Deep Belief Network (DBN), and restricted Boltzmann machine (RBM)-based 1SVM methods. However, this detector is verified using routine blood tests samples from two hospitals in Brazil and Italy; a large dataset is needed to verify the generalization of this approach. In^15^, an intelligent framework based on deep learning and cloud computing is presented to identify potential violations of COVID norms in workplaces. To this end, this approach employs Closed Circuit Television (CCTV) cameras and webcams installed in workplaces. This approach can detect two types of violations: mask-wearing and physical distancing between employees. Results based on a video of almost eight hours demonstrated that this framework achieved 98% accuracy. However, this approach can be improved by including other COVID norms and tracking the location of employees’ movement after office hours. Essentially, AI presents relevant support to predict pandemics and take early measures to mitigate the negative consequences. Much research has been done recently on developing data-driven techniques to combat the COVID-19 pandemic. For example, see some relevant review articles on detection and forecasting of COVID-19^16–19^.

Efforts devoted to mitigating the effects of COVID-19 transmission have been conducted since its appearance in December 2019. Recently, there have been many studies conducted to understand and manage the COVID-19 pandemic by developing several techniques for different applications, such as wearing mask detection^20^, COVID-19 spread forecasting^21^, and chest X-rays diagnosis^22^. Wearable technologies have been recently demonstrated promising solutions to aid in mitigating infectious diseases, such as COVID-19. In^23^, the authors presented an overview of different wearable monitoring devices and respiratory support systems that are used for assisting patients infected with COVID-19. Even with the promising potential of wearable technologies for slow downing the spread of COVID-19, however, their utilization is still limited due to several restrictions, such as data privacy and cyber-attacks. AI-enabled systems have also been designed to detect people that are not wearing any facial masks to mitigate the propagation of COVID-19 spread. For instance, in^24^, a vision-based deep learning approach has been proposed for facial mask detection in a smart city network. Results revealed that this approach achieved 98.7% accuracy in discriminating people with facial masks from people without masks. However, to guarantee sufficient monitoring, a large number of cameras is needed to cover the whole monitored city, which is not easy to get and also has an economic burden. Accurate forecasting of COVID-19 cases is essential to help mitigate and slowdown COVID-19 transmission^25–27^. In^4^, the authors present a comparative study between eight machine learning models to forecast COVID-19, such as logistic regression, Restricted Boltzmann Machine, convolutional neural networks, and support vector regression(SVR). They used time-series data for confirmed and recovered COVID-19 cases from seven countries, including Brazil, India, and Saudi Arabia, recorded from January 22, 2020, to September 06, 2020. It has been shown that machine learning models can track the future COVID-19 trend even in the presence of a relatively small-sized dataset. The convolutional neural networks-Long short-term memory LSTM-CNN showed high performances with an averaged mean absolute percentage error (MAPE) of around 3.718%, because of its ability to learn higher-level features. The study in^28^ used machine learning to predict weekly cumulative COVID-19 cases recorded in the USA and Germany. The first 18 weeks are employed for models construction, and 17 weeks after 18/09/2020 are used for testing. Results showed that the SVR delivers the best prediction accuracy compared to Random Forest (RF), Linear Regression (LR), and Multilayer perceptron (MLP) in terms of Root Mean Square Error (RMSE) and MAPE metrics. Specifically, the SVR model achieved an averaged MAPE of 0.1162%. However, in this study, only weekly predictions are considered, and daily COVID-19 cases predictions, which are important to short-term decision making, are ignored. In^29^, authors focused on forecasting the future number of COVID-19 in the next 60 days for the confirmed, recovered, and death cases in the 16 high impacted countries. To this end, they considered a Seasonal Auto-Regressive Integrated Moving Average (SARIMA) and Auto-Regressive Integrated Moving Average (ARIMA). The result reveals that the SARIMA model is more realistic than the ARIMA model in this study. The study in^30^ employed an autoregression model utilizing Poisson distribution called Poisson Autoregression(PAR) to predict the confirmed and recovered cases of COVID-19 in Jakarta. Results showed that this approach provides acceptable forecasting accuracy with an MPAE value lesser than 20%. This approach showed better performance compared to conventional methods, including ARIMA, Exponential Smoothing, BATS, and Prophet. However, the Poisson Autoregression approach’s prediction quality still requires more improvement to reach a satisfactory prediction performance. Similarly, in^31^ the ARIMA model has been employed for daily prediction of COVID-19 spread in Italy, Spain, and France based on data collected between 21/02/2020-15/04/2020. This approach showed satisfactory prediction performance by achieving an average MAPE of 5.59%. Although ARIMA models can provide suited prediction accuracy of data with regular trends, they are limited in extracting only linear relationships within the time series data. The study in^32^ investigated linear regression and Polynomial regression for forecasting the spread of COVID-19 cases in India using data from March 12 to October 31, 2020. Forecasting results showed that the Polynomial model with 2 degrees outperformed the linear regression model by achieving an averaged MAPE of 13.3%. However, the forecasting accuracy can be improved by using a large dataset and fine-tuning the model parameters. The work in^33^ considered SVM and Multilayer Perceptron (MLP) methods to predict confirmed COVID-19 cases using data recorded in Brazil, Chile, Colombia, Mexico, Peru, and the US from 1/22/2020 to 5/25/2020. This study reported that the MLP model outperformed the SVM by providing an averaged MAPE of 17%. The hyperparameters were optimized via a tabu list algorithm. Another study^34^ presented a comparison of four methods, ARIMA, ANN, LSTM, and CNN, to predict the COVID-19 spread based on data available from March 12 to October 31, 2020. The CNN model outperformed the other models by achieving an averaged MAPE of 3.13%. However, the models were trained using small-sized data, making it difficult to get accurate models for forecasting the future trends of COVID-19 spread. In^35^, the paper focuses on predicting future COVID-19 confirmed and death cases in nine high affected countries from January 22, 2020, till December 13, 2020. Four factors are used as input variables, including vaccination, weather conditions, malarial treatments, and average age, to predict COVID-19 spread. This study reported that the Multilayer perceptron (MLP) model provided satisfactory forecasting accuracy. Authors in^36^ proposed a cloud-based short-term forecasting model to predict the number of COVID-19 confirmed cases for the next seven days. Results indicate the importance of the cloud-based short-term forecasting model in decision-making to prepare the needed medical resources. In^37^, a modified version of LSTM (M-LSTM) has been introduced to forecast the COVID-19 outbreak in nine countries from three continents. Specifically, the authors used data from January 22 till July 30, 2020, for the train set and last month, August, for the test. It has been shown that the M-LSTM is the winner model among other investigated models. In^38^, LSTM and gated recurrent unit (GRU) deep learning models have been applied to forecast COVID-19 confirmed cases and deaths in Saudi Arabia, Egypt, and Kuwait from 1/5/2020 to 6/12/2020. In this study, LSTM with a single layer exhibited the best forecasting of confirmed cases with an average MAPE of 0.6203%. The authors in^39^ implemented an approach for forecasting COVID-19 by combining Graph Neural Networks (GNNs) within the gates of an LSTM to enable exploiting temporal and spatial information in data. Results based on data of 37 European nations show better performance compared to state-of-the-art methods by reaching a mean absolute scaled error (MASE) value around 0.27. However, this approach can be improved by considering other pertinent factors like poverty rates, hospital capacity, and age demographics. Further, authors in^40^ considered four machine learning models (i.e., Linear Regression (LR), Least Absolute Shrinkage, and Selection Operator (LASSO), Random Forest (RF), and Ridge Regression (RR)) to forecast future COVID-19 cases. The result shows that the RF outperformed the other models. Two machine learning models, namely Neural Network Time Series (NAR-NNTS) and Nonlinear Autoregressive (NAR), were evaluated by^41^ to forecast COVID-19 cases. Results indicate the outperformance of the NAR-NNTS model compared to the NAR model. In^42^, four regression models, ARIMA, MLP, LSTM, and feedforward neural network (FNN), are considered to predict COVID-19 spread. It has been shown that the LSTM model reached the best forecast accuracy in this study. In^43^, the aim is to predict confirmed and deaths cases recorded in Iran and Australia by considering one, three, and seven past-day ahead in the next 100 days. This study applied six models: LSTM, GRU, and Convolutional LSTM with their bidirectional extension. The results showed that the bidirectional models achieve better performance than non-bidirectional most of the time. This could be attributed to forward and backward data processing in bidirectional models, which allow better learning temporal-dependencies in COVID-19 data. In^44^, six models, including Susceptible-Infected-Recovered, Linear Regression, Polynomial Regression, and SVR and LSTM, are compared in forecasting COVID-19 cases in Saudi Arabia and Bahrain. Results reveal that SVR provides the best forecasting when using confirmed COVID-19 cases data from Saudi Arabia, and LR outperforms the other models when using Bahrain confirmed cases data.

Accurate forecasting of COVID-19 spread is a key factor in mitigating this pandemic’s transmission by providing relevant information to help hospital managers in decision-making and appropriately managing the available resources and staff. In the presence of small-sized COVID-19 data, our objective is to present shallow and efficient machine learning methods to forecast future trends of COVID-19 spread. The most common machine learning approaches for COVID-19 time series forecasting rely only on the actual data point in the forecasting process and ignores the information from past data. Thus, The overarching goal of this study is to take into account information from the actual and past data in developing efficient machine learning models to accurately forecast COVID-19 spread. Specifically, this study investigates the forecasting ability of the optimized GPR, a kernel-based machine learning method, in forecasting the COVID-19 time series. This choice is motivated by the desirables features of the GPR model, including its simple and flexible construction using the mean and covariance functions, its ability and superior nonlinear approximation, and the possibility to explicitly provide a probabilistic representation of forecasting outputs^8,45^. The contributions of this paper are summarized in the following key points.

- Firstly, we employed Bayesian optimization (BO) to tune the Gaussian process regression (GPR) hyperparameters to develop an efficient GPR-based model for forecasting the recovered and confirmed COVID-19 cases in two highly impacted countries, India and Brazil. We compared the performance of the Optimized GPR with 16 models, including Support vector regression with different kernels, GPR with different kernels, Boosted trees, Bagged trees, Random Forest, and eXtreme Gradient Boosting (XGBoost). The daily records of confirmed and recovered cases from Brazil and India are adopted in this study. The k-fold cross-validation technique has been considered in constructing these models based on the training data. Three statistical criteria are used for the comparison. The results showed that the optimized GPR model exhibited a superior prediction capability over the other models.
- However, machine learning models do not consider the time dependency in the COVID-19 data series. The time dependency in COVID-19 data can be captured by incorporating lagged data in designing the considered ML models. Meanwhile, considering information from past data is expected to improve the ML models’ capabilities to effectively follow the trend of future COVID-19 data. Here, we evaluated the potential of incorporating dynamic information to further enhance the forecasting performance of the investigated ML models. The results clearly reveal that the lagged data contribute significantly to improved prediction quality of the ML models and highlight the superior performance of the dynamic OGPR.
- Additionally, after showing the necessity of including information from past data to enhance the investigated machine learning models, we assessed the importance or contribution of the included features to the COVID-19 prediction quality. Importantly, we applied the RF algorithm to identify variable contribution or importance for predictive ML models. Generally speaking, this step is essential to design parsimonious models by ignoring unimportant features.
- Finally, we provided the confidence level of the predicted results based on the dynamic OGPR model and showed that the predictions are within the 95% confidence interval.

Of course, we conclude that the dynamic OGPR model is an efficient forecasting approach and can predict confirmed and recovered COVID-19 times series data with high accuracy.

The remaining of this study is structured as follows. The second Section presents the used COVID-19 datasets, provides a brief description of the GPR model and the BO algorithm. The results and discussions were given in the third section to show model performances and comparisons. The conclusions are outlined in the fourth Section.

## Methodology

**Figure 1:**
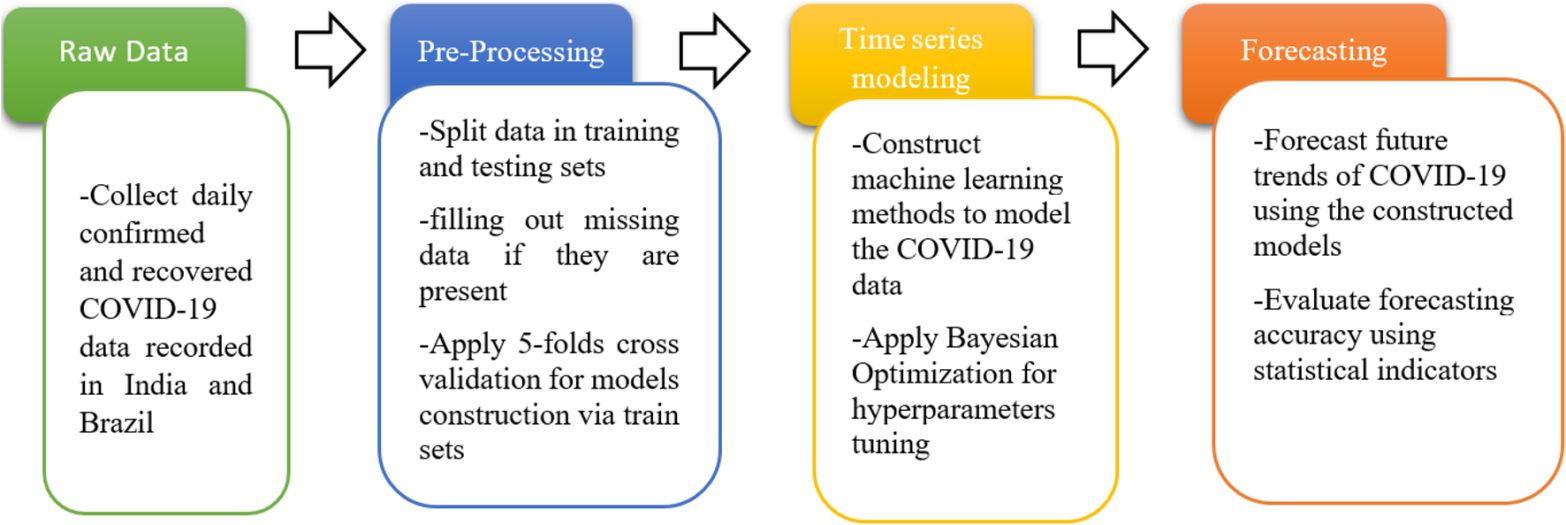
Schematic presentation pf the used machine learning-based forecasting framework.

The overarching goal of this study is to provide accurate forecasting of the recovered and confirmed COVID-19 cases in two highly impacted countries, India and Brazil. In total, eighteen machine learning models have been investigated and compared against each other for COVID-19 time-series forecasting. The general framework adopted in this study is depicted in Fig. 1. At first, We feed the model with training data to find the parameters that minimize the loss functions in training. Specifically, we used the Bayesian Optimization algorithm, a powerful tool for the joint optimization of design choices, to hyperparameters tuning. After that, the constructed models are used to forecast the future trend of COVID-19 spread. The model’s accuracy will be checked by comparing measured data to forecasted data via the score indicators.

### Data description

**Figure 2:**
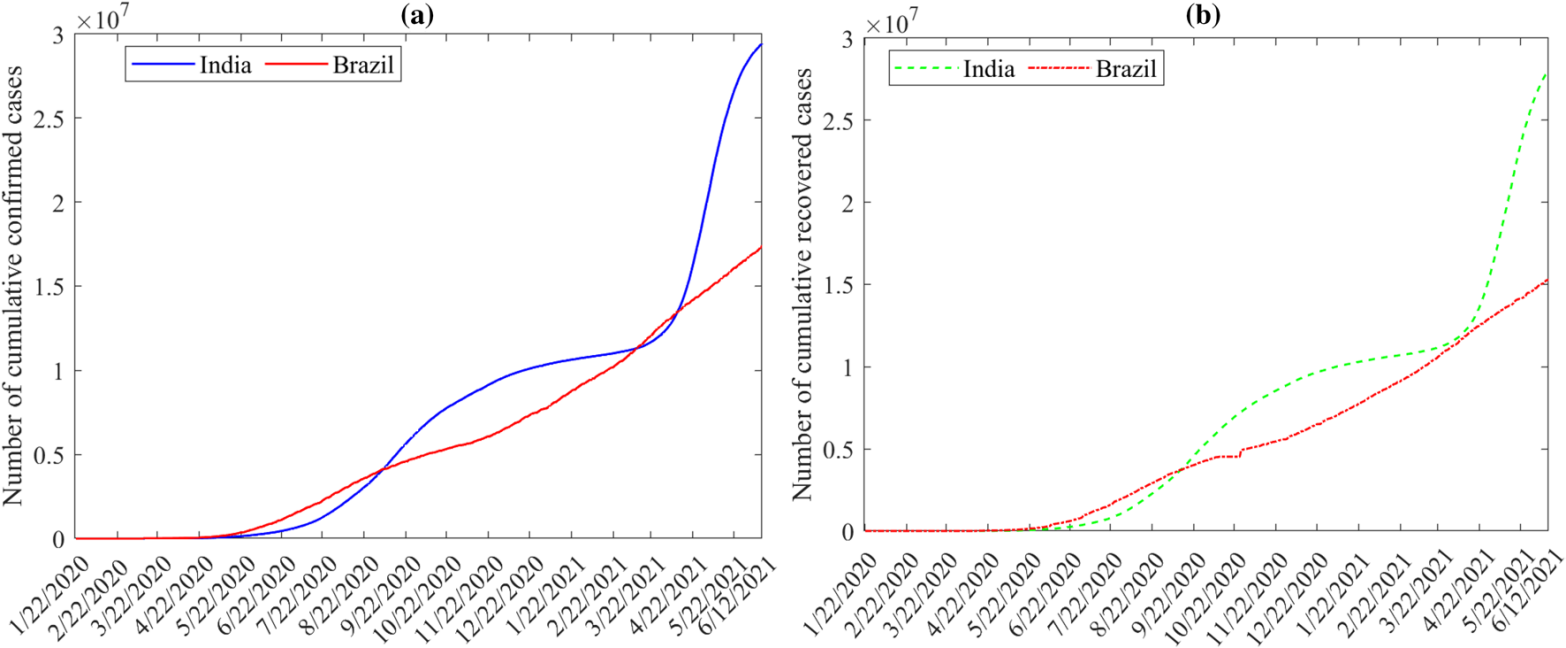
The number of (**a**) confirmed and (**b**) recovered COVID-19 cases from January 22, 2020, through June 12, 2021, in Brazil and India.

Here, daily confirmed and recovered COVID-19 data from two highly impacted countries, India and Brazil, are utilized to evaluate the forecasting capacity of the 14 investigated data-based methods. The daily record of cumulative confirmed and recovered cases of COVID-19 from the first case, in India and Brazil on the 30th of January and 26th of February 2020, are available in (https://github.com/CSSEGISandData/COVID-19). The dataset automated update for delayed data in the website without any missing value. Figure 2a–b displays the confirmed and recovered COVID-19 cases dataset used in this study. We observe that India has the highest number of confirmed cases. Considering the population in each country, India is receiving the most considerable impact from COVID-19. On the other hand, India shows rapid growth in recovered cases, indicating their prompt and effective response to this public health event.

Table 1 list the descriptive statistics of the used COVID-19 time-series dataset. We can conclude from Table 1 that these datasets are non-Gaussian distributed.

**Table 1:** Summary of the used COVID-19 time-series dataset.

Series	Q1	Median	Mean	Q3	Std	skewness	kurtosis
Confirm India	161736	6433806	7146174.35	10820333.5	7549037.149	1.169865194	3.997238807
Confirm Brazil	425029.5	4847092	5690061.496	9447165	5246461.833	0.61590352	2.19566924
Recovered India	69334.5	5389892	6454191.404	10516698.5	6870596.966	1.117660039	3.972183915
Recovered Brazil	172125.5	4299659	4965903.071	8412570	4689476.608	0.604219818	2.150912456

**Figure 3:**
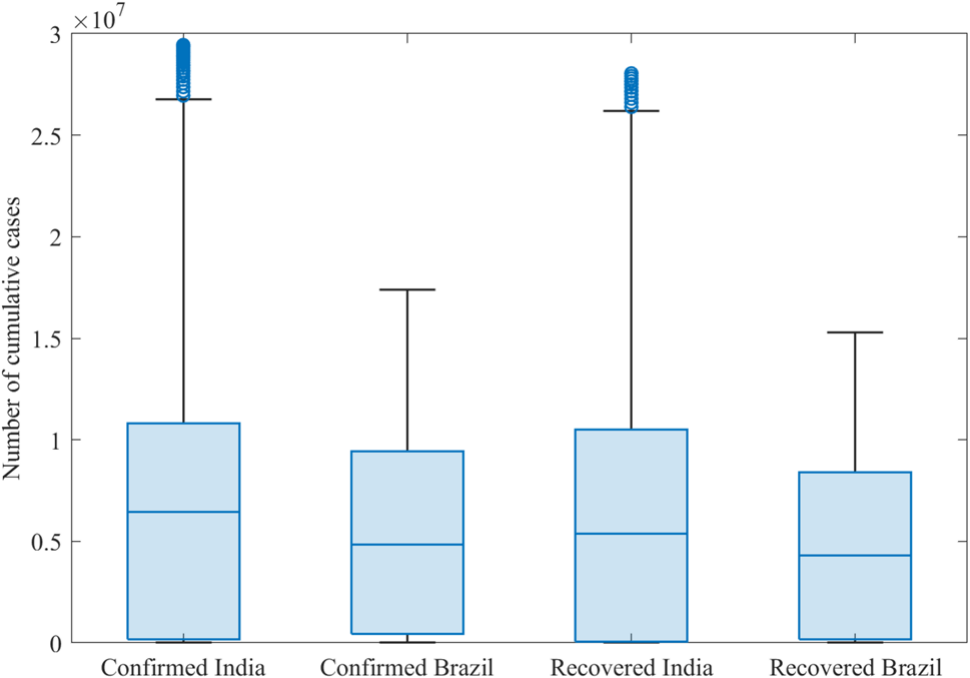
Boxplots of the daily number of confirmed and covered COVID-19 time-series datasets in India and Brazil.

Figure 3 illustrates boxplots of the confirmed and recovered COVID-19 cases recorded in India and Brazil. We observe that the distributions of the recorded confirmed and recovered cases are heavily right-skewed.

**Figure 4:**
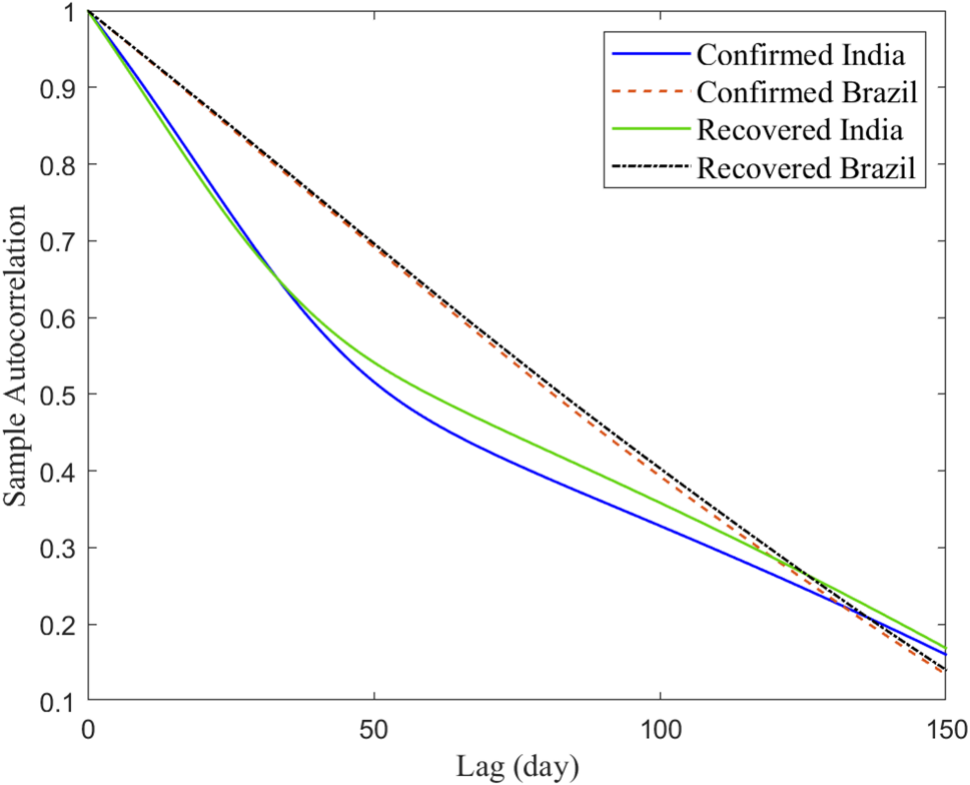
Sample Autocorrelation functio of confirmed and covered COVID-19 time-series datasets in India and Brazil.

For the COVID-19 time series data in Fig. 2a–b, the the autocorrelation function (ACF) are shown in Fig. 4. The ACF measures the similarity between $y_{t}$ and $y_{t+k}$, where $k=0, \dots , l$ and $y_{t}$ is the investigated COVID-19 times series data^46^. In other words, ACF quantifies the self-similarity of the univariate time-series data over different delay times. Mathematically, the ACF of a signal $y_t$ is defined as^46^,

1 $$\begin{aligned} {\rho _k} = \frac{{{\mathop {\mathrm {cov}}} \left( {{y_t},{y_{t - k}}} \right) }}{{\sqrt{{\mathop {\mathrm {var}}} \left( {{y_t}} \right) {\mathop {\mathrm {var}}} \left( {{y_{t - k}}} \right) } }}\ \end{aligned}$$

The ACF of the data in Fig. 2a–b provides some relevant information about the time-dependence and process structure in COVID-19 data points. From Fig. 4, we first observe that there is short-term autocorrelation in these COVID-19 datasets. Also, we observe the similarity between the ACFs of confirmed and recovered time-series in each country. The third observation is that the fluctuation of India’s data recorded is relatively different from Brazil’s data. This could be attributed to the high spread of COVID-19 in INDIA compared to India. Regarding the population in every country, India is getting the most significant impact from COVID-19.

### GPR model

The GPR, a supervised nonparametric (Bayesian) machine learning method, can flexibly model complex nonlinear relationships between input and output variables^47^. GPR is an effective kernel-driven approach to learn implicit correlations among various variables in the training set, making GPR especially suitable for challenging nonlinear prediction^48^. Importantly, GPR, a probabilistic-based nonlinear regression approach, owns desirables characteristics, including the capability for handling large dimensionality, small-sized data, and complex regression problems^47^.

For a prediction problem, the output ${\mathbf {y}}$ of a function ${\mathbf {f}}$ at the input ${\mathbf {x}}$ in GPR is expressed as,

2 $$\begin{aligned} {\mathbf {y}}_{i} = f({\mathbf {x}}_{i}) + \mathbf {\varepsilon } _{i}. \end{aligned}$$

where $\mathbf {\varepsilon }\sim {\mathcal {N}}(0, \sigma ^{2}_{\varepsilon })$. In GPR, the term, *f*(*x*), is assumed to be a random variable that is distributed according to a particular distribution. Indeed, observing the output of the function at various input points could reduce the uncertainty regarding *f*. The observations are always tainted with a noise term $\varepsilon$ that reflects their inherent randomness.

Assume ${\mathcal {D}} = \lbrace (x_{i}, y_{i})\rbrace _{i=1}^{n}$ is the input-output measurements and $f(\cdot )$ to be approximated and assumed following a Gaussian process. For the sake of simplicity, let assume that $x_{i}$’s and $y_{i}$ ’s are scalar observations while $\varepsilon _{i}$ ’s are independent and identically distributed random noises following the normal distribution with mean value ${\bar{\varepsilon }}_{i}=0$ and variance $\sigma ^{2}$.

Let’s consider the measured $y_{i}$ values $[y_{1}, y_{2}, {\dots }\, y_{n}]^{\top }$ are finite values of the function $f(\cdot )$ contaminated with noises. Thus, $y_{i}$ ’s follow a joint Gaussian distribution:

3 $$\begin{aligned} {\mathbf {y}} = [y_{1}, y_{2}, {\dots }\, y_{n}]^{\top } \sim {\mathcal {N}}({\mathbf {m}}({\mathbf {x}}), {\mathbf {K}}+\sigma ^{2}{\mathbf {I}}), \end{aligned}$$

where ${\mathbf {m}}({\mathbf {x}}) = [m(x_{1}), m(x_{2}), {\dots }\, m(x_{n})]^{\top }$ represents the mean vector $m(\cdot )$, ${\mathbf {I}}$ refers to the identity matrix, and ${\mathbf {K}}$ denotes the $n\times n$ covariance matrix with $(i,j){\rm{th}}$ element ${\mathbf {K}}_{ij} = k(x_{i}, x_{j})$. For a GPR model, $k(x_{i}, x_{j})$ is usually termed a kernel function^49^.

The optimized kernel parameters are achieved by maximization of the following likelihood.

4 $$\begin{aligned} {\varvec{\theta }}_{\mathrm {opt}} = \mathop {\arg \,max}\limits _{\varvec{\theta }} L(\varvec{\theta }) \end{aligned}$$

where $\mathbf {\theta } = [\theta _{1}, \theta _{2}, \, \dots ]$ refers to kernel parameters, the mean values *m*(.) are chosen to be zero, and

5 $$\begin{aligned} L(\varvec{\theta }) = \frac{1}{\sqrt{(2\pi )^{n}\!| {\mathbf {K}}+\sigma ^{2}{\mathbf {I}} |}} \exp \!\left( {-\frac{1}{2} ({\mathbf {y}}^{\top }({\mathbf {K}} +\sigma ^{2}{\mathbf {I}}){\mathbf {y}})}\right) . \end{aligned}$$

In this study, Bayesian optimization will be applied to determine the optimal GPR hyper-parameters via the maximization of the marginal likelihood in (4) with respect to $\theta$^50^.

Let $x_{*}$ is a new input, then the predictive mean and variance associated with ${\hat{y}}_{*}=f(x_{*})=f_{*}$ are respectively expressed as follows:

- the mean value
  6 $$\begin{aligned} {\hat{y}}_{*} = {\mathbf {k}}_{*}^{\top }({\mathbf {K}} + \sigma ^{2} {\mathbf {I}})^{-1}{\mathbf {y}} \end{aligned}$$
- and variance
  7 $$\begin{aligned} {\Sigma }_{*} = k_{**} - {\mathbf {k}}_{*}^{\top }({\mathbf {K}}+\sigma ^{2} {\mathbf {I}})^{-1}{\mathbf {k}}_{*} \mathrm {.} \end{aligned}$$
- and ${\mathbf {y}}_{*}$ follows a conditional distribution:
  8 $$\begin{aligned} y_{*}|{\mathbf {y}} \sim {\mathcal {N}}({\hat{y}}_{*}, {\Sigma }_{*}) \end{aligned}$$

where $${\mathbf {K}}={\mathbf {k}}({\mathbf {x}}, {\mathbf {x}})$$ refers to the covariance matrix of training data; $$\mathbf {K_{**}}={\mathbf {k}}({\mathbf {x}}_{*}, {\mathbf {x}}_{*})$$ represents the covariance of testing data, and $${\mathbf {K}}_{*}={\mathbf {k}}({\mathbf {x}}, {\mathbf {x}}_{*})$$ represents the covariance matrix obtained using the training and test dataset.

The GPR predicted output value for a given test input ${\mathbf {x}}$ is $\mathbf {{\overline{f}}}^{*}$. In addition to the predicted output, GPR can provide a confidence interval (CI) to assess the reliability of the prediction, which can be computed using the variance $cov(\mathbf {{\overline{f}}}^{*})$. For example, the 95% CI is computed as^51^,

9 $$\begin{aligned} CI=\bigg [ \mathbf {{\overline{f}}}^{*} - 2 \times \sqrt{cov(\mathbf {{\overline{f}}})},~ \mathbf {{\overline{f}}}^{*}+2 \times \sqrt{cov(\mathbf {{\overline{f}}})}\bigg ]. \end{aligned}$$

For more details about GPR model, see^52,53^.

### Bayesian optimization of model parameters

Various machine learning methods, including GPR and ensemble models, include many hyperparameters to be chosen (e.g., kernel types in GPR and parameters). Essentially, the selected values of hyperparameters highly impact the performance of machine learning models^54^. Accordingly, several optimization methods to search for the best hyperparameter, including grid search, random search, and Bayesian Optimization (BO), are reported in the litterature^55^. The Grid search essentially made a grid of the search space and then evaluated each hyperparameter setting at the points we introduced for as many dimensions as necessary^56^. On the other hand, Random search uses a random combination of a range of values and compares the result in each iteration, but this method will not guarantee to get the best hyperparameter combination^56^. This study employed the BO procedure, which is frequently applied in machine learning to find the optimal values of hyperparameters. This study applied the Bayesian optimization algorithm to find the optimal hyperparameters of four investigated methods: SVR, GPR, Boosted trees, and Bagged trees. Notably, the BO algorithm is an efficient and effective global optimization approach that is designed based on Gaussian processes and Bayesian inference^50^. Crucially, Bayesian Optimization can bring down the time spent to get to the optimal set of parameters by considering the past evaluations when choosing the hyperparameters set to evaluate next^57^.It could be employed to optimize functions with unknown closed-form^58^. Although, unlike grid search, BO can find the optimal hyperparameters with fewer iterations.

The essence of the BO algorithm is to construct a probabilistic proxy model for the cost function based on outcomes of historical experiments as training data. Essentially, the proxy model, such as the Gaussian process, is more inexpensive to compute, and it gives sufficient information on where we should assess the true objective function to obtain relevant results. Let’s consider *m* hyperparameters ${\mathbf {P}}={{\mathbf {p}}_{1}, \dots , {\mathbf {p}}_{m}}$ to be tuned. The aim is to determine

10 $$\begin{aligned} {\mathbf {P}}^{*}= \mathop {\arg \,min}\limits _{{\mathbf {P}}} {{\mathbf {g}}} ({\mathbf {P}}|\{(x_{i},y_{i})\}_{i=1}^{n}), \end{aligned}$$

where ${\mathbf {g}}$ is a cost function. The whole optimization procedure is controlled via a suitable acquisition function (AF) that defines the following set of hyperparameters to be assessed. Crucially, any acquisition function requires adjusting within exploration and exploitation. Generally speaking, exploration is an area search with high uncertainty, where we expect to discover a new set of parameters that enhance the model’s prediction accuracy. At the same time, exploitation refers to an area search nearby to already computed high estimated values^59^.

**Figure 5:**
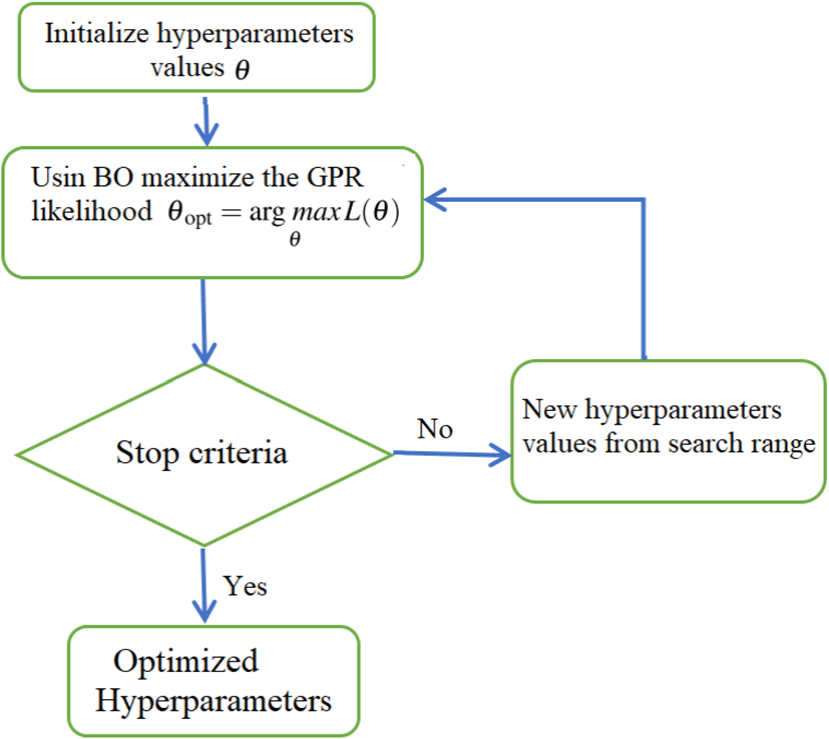
BO-based optimized GPR procedure.

In this study, the BO algorithm is employed to find the hyperparameters of the GPR model, the SVR, and ensemble learning models. The optimization procedure is performed during the training stage based on the training data, as shown in Fig. 5. At each iteration, the mean squared error (MSE) between the actual COVID-19 data and the estimated GPR data using the values of the hyperparameters determined by BO. This procedure is repeated until the MSE converges to a small value, close to zero.

### Alternative models for Comparison

In this study, we investigated the performance of the OGPR and compared its forecasting accuracy with the set of machine learning-based forecasting models listed in Table 1. In short, a total of seventeen forecasting methods are applied to predict COVID-19 time-series data: 6 SVR methods^48,60^, 4 GPR methods, 2 ensemble learning techniques (i.e., BT, BS, RF and XGBoost)^61–63^ and six SVR models^47^, and 3 optimized methods.

**Table 2:** Forecasting methods investigated in this study.

Model approach	Model name	Model description	Kernel function$^{(1)}$
Support Vector Regression (SVR)	SV_L	SVR with the Linear kernel	${x^{T}_{i}}x_{j}$
	SVR_Q	SVR with the Quadratic kernel	$(1+{x^{T}_{i}}x_{j})^2$
	SVR_C	SVR with the Cubic Kernel	$(1+{x^{T}_{i}}x_{j})^3$
	SVR_FG	SVR with the Fine Gaussian kernel	$e^{(-\frac{\sqrt{p}}{4}||x_{i}-x_{j}||^2)}$
	SVR_MG	SVR with the Medium Gaussian kernel	$e^{(-\sqrt{p}||x_{i}-x_{j}||^2)}$
	SVR_CG	SVR with the Cubic Gaussian kernel	$e^{(-4\sqrt{p}||x_{i}-x_{j}||^2)}$
Gaussian Process Regression (GPR)	GP_RQ	GPR with the Rational Quadratic kernel	${\sigma ^{2}_{f}}(1+\frac{r^{2}}{2\alpha \sigma ^{2}_{l}})^{-\alpha }$
	GP_SE	GPR with the Squared Exponential kernel	${\sigma ^{2}_{f}}e^{(\frac{r^{2}}{2\sigma ^{2}_{l}})}$
	GP_M52	GPR with the Matern 5/2 kernel	${\sigma ^{2}_{f}}(1+\frac{\sqrt{5r}}{\sigma l}+\frac{5r^2}{3\sigma ^{2}_{l}})e^(\frac{\sqrt{5}r}{\sigma l})$
	GP_Exp	GPR with the Exponential kernel	${\sigma ^{2}_{f}}e^(\frac{r}{\sigma l})$
Ensemble Learning (EL)	BST	Boosted Trees	
	BT	Bagged Trees	
	RF	Random Forest	
	XGBoost	eXtreme Gradient Boosting	
Optimised models	OSVR	Optimized SVR	
	OGPR	Optimized GPR	
	OEL	Optimized EL	

${}^{(1)} r=\sqrt{(x_{i}-x_{j})^1(x_{i}-x_{j})}$ in the GPR-based kernel function.

#### SVR models

Support Vector regression is another efficient assumption-free approach that possesses good learning ability through kernel tricks. The essence of SVR is to map the train data to a higher dimension then linear regression is performed in this feature space. In short, SVR can efficiently deal with nonlinear regression via the so-called kernel trick by mapping the input features into high-dimensional feature spaces^64,65^. It is designed using the concept of structural risk minimization. Moreover, SVR models proved to be efficient in the presence of limited samples^66^. Additionally, SVR has been broadly applied in various applications, including wind power forecasting^67^, fault detection^8^, and solar irradiance prediction^48^. This study built six SVR models using different kernels and an optimized SVR using Bayesian optimization (Table 2).

#### Boosted tree model

Boosted is an ensemble machine learning model built based on the statistical learning theory. The essence of the boosted tree is to optimize the prediction quality of conventional regression methods by using an adaptive combination of weak prediction models^68^. Moreover, it employs an aggregate model to obtain a smaller error than those obtained by individual models. Compared to other ensemble models, like bagging and averaging models, boosting is matchless because of the sequentiality^63,68,69^.

#### Bagged tree model

The bagged tree (BT) is an ensemble machine learning model; also, it is called bootstrap aggregating. Essentially, BT merges the bagging procedure and decision trees to improve prediction efficiency^61^. Specifically, The bagged model generates multiple samples via bootstrap sampling from the original dataset, builds multiple distinct decision trees, then aggregates their prediction outputs together^70^. Accordingly, the prediction error of the decision trees will be reduced, and substantially the overfitting problem in a single tree is bypassed^71,72^.

#### Random forest

RF is also within the ensemble learning family that uses several weak learners to build a more efficient joint model^73^. In the RF model, decision trees are used as a base learner. The RF repeatedly builds regression trees based on the training data. In boosting, each new training set is sampled with replacement from the original training set by using the bootstrap technique. However, the strategy for node selection in RF is different by randomly selecting a subset from the current feature set and then selecting one optimized feature in the sub-feature set. It has been widely exploited in different applications related to classification and regression problems.

#### XGBoost model

Extreme Gradient Boosting algorithm (XGBoost) is an efficient ensemble learning algorithm that can handle missing values and combine a set of weak predictors for building a more effective one^74^. It can be used for classification and prediction problems. XGBoost can reduce the loss function by employ the gradient descent method to determine the objective function optimization. Especially, XGBoost will avoid the overfitting in the model by relying on a set of learners to build a robust model that also helps minimize the running time. XGBoost is flexible and efficient and is adopted in many winning data mining competitions^75^.

### Evaluation metrics

In this study, we assess the accuracy of the forecasting models using three metrics: root mean square error (RMSE), mean absolute error (MAE), and mean absolute percentage error (MAPE).

11 $$\begin{aligned} RMSE = \sqrt{\frac{1}{n}\sum _{t=1}^{n}(y_{t}-{\hat{y}}_{t})^{2}}, \end{aligned}$$

12 $$\begin{aligned} MAE = \frac{\sum _{t=1}^n\left| y_t-{\hat{y}}_{t}\right| }{n}, \end{aligned}$$

13 $$\begin{aligned} MAPE = \frac{100}{n} \sum _{t=1}^{n}\bigg |\frac{y_{t}-{\hat{y}}_{t}}{y_{t}}\bigg |\%, \end{aligned}$$

where $y_{t}$ is the number of COVID cases, ${\hat{y}}_{t}$ is its corresponding forecasted COVID cases, and *n* is the number of records. Lower RMSE, MAE, and MAPE values would imply better precision and forecasting quality.

### Forecasting framework

**Figure 6:**
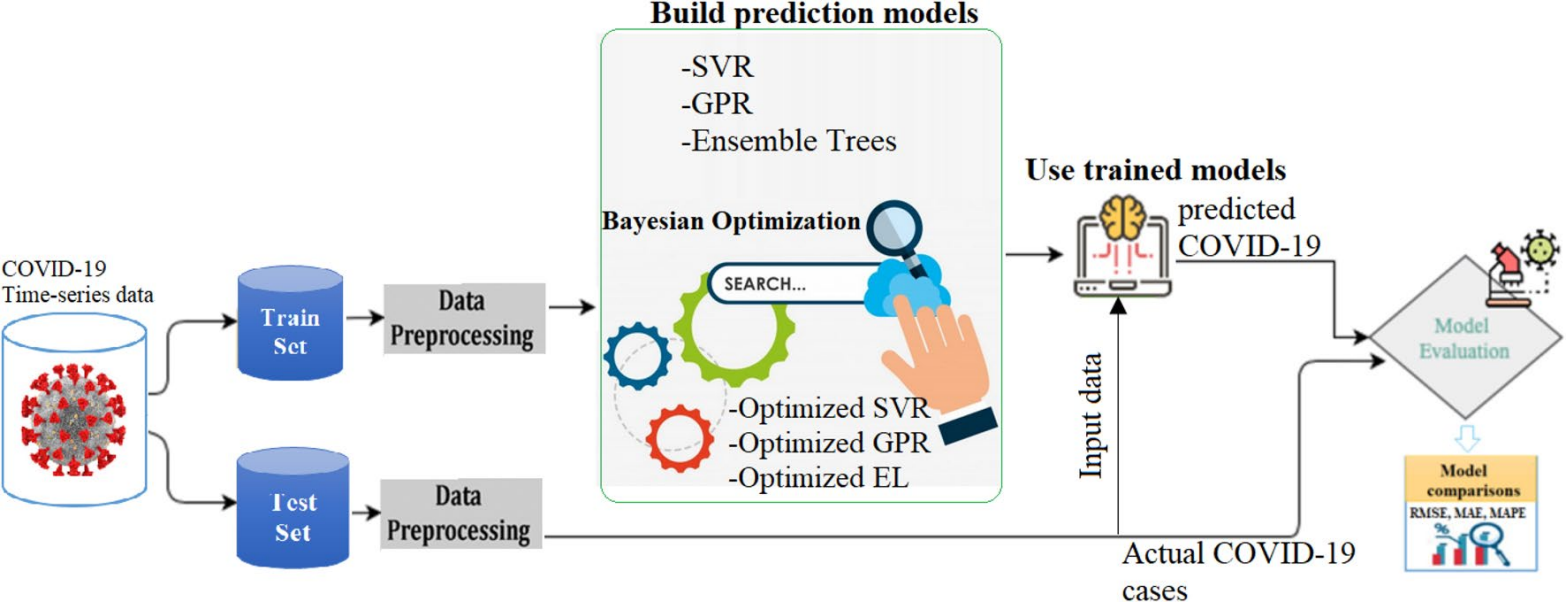
Illustration of the used forecasting framework.

The general procedure performed in this study to forecast COVID-19 cases is represented in Fig. 6. Firstly the daily recovered and confirmed time-series data are split into training subsets. All models are trained using the training set and evaluated using the testing set. The best forecasting model is indicated by three statistical criteria, namely RMSE, MAE, and MAPE.

## Results and discussion

### Static prediction models

The COVID-19 time series used in this study is free from missing values. We first split these data into training sub-data and testing sub-data. The training data used to construct each model includes confirmed and recovered cases from January 22, 2020, to June 5, 2021. We used seven days for the testing period from June 6, 2021, to June 12, 2021. Here, we mean by static prediction models, the models that predict COVID-19 spread a given time point without considering information from past data.

**Figure 7:**
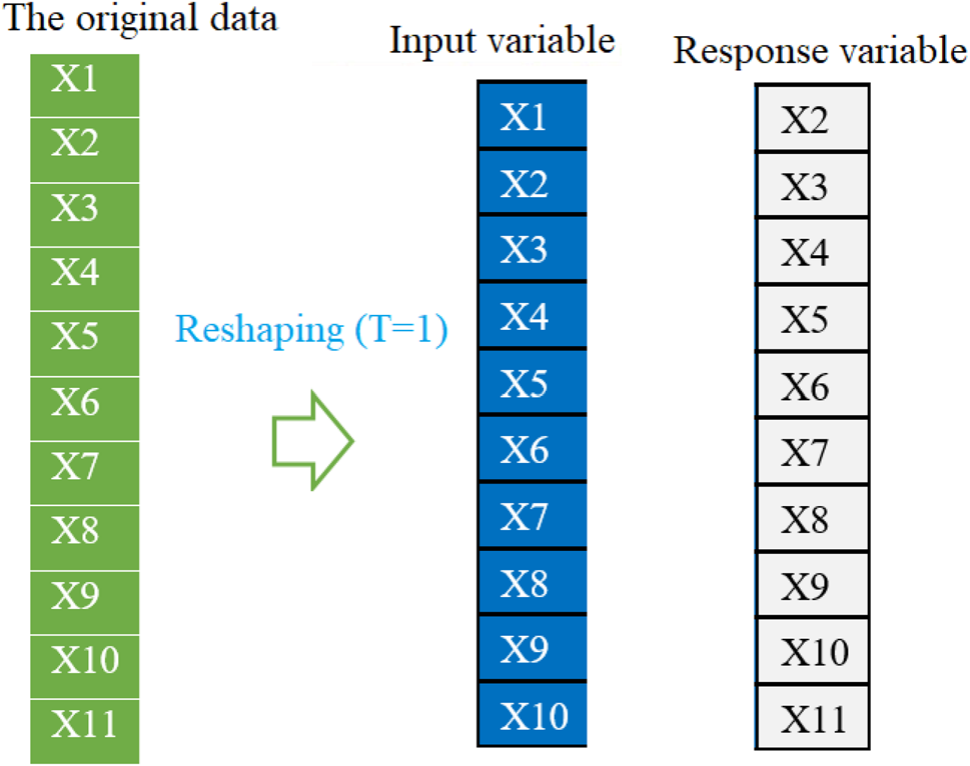
Procedure to restructure univariate COVID-19 time-series data to look like input-output data.

Firstly, we need to transform the time-series forecasting problem into a supervised learning problem to apply the investigated machine learning models. In other words, univariate COVID-19 time-series data will be preprocessed to get pairs of input and output data points. In supervised learning, the models first learn the mapping between the input and output variables based on training data, and then they can be used to predict the output from the input test data. We can structure the data to look like input-output data. This can be done by utilizing previous data points as input variables and use the next data point as the response variable (see Fig. 7). We can see from Fig. 7 that shifting the series forward one step allows us to use the previous observations to predict the value at the next time step.

The k-fold cross-validation technique has been considered in constructing these models based on the training data as recommended in^76,77^. Specifically, we applied a 5-fold cross-validation technique in training the investigated models. This permits assessing the models’ robustness, exploiting the whole training dataset, and helps avoid overfitting. In the training stage, the considered models are constructed by finding the appropriate values of hyperparametrs that produce high prediction accuracy. In the BT model, we used 30 trees with a minimum leaf size of 8. Similarly, in BST, 30 trees are used as based learners with a minimum leaf size of 8 and a learning rate of 0.1. We used the SVR model with Kernel scale: 0.25, box constraint: 6.534, and Epsilon: 1.3156. Here, GPR models with four different kernels are considered. The values of Sigma and kernel scales of GPR$_{SE}$, GPR$_{RQ}$, GPR$_{M25}$, and GPR$_{Exp}$ are respectively (5102.98, 508349.66), (51029.88, 5083496.65), (51029.87, 5083496.65), and (51029.92, 5083496.65). For the RF model, 1000 trees are used in the forest, and ’max_features=1’ is chosen to consider only one feature to find the best split, and ’random_state=1’ is selected for controlling both the randomness of the bootstrapping of the samples used when building trees. For the XGBoost model, the values of the used hyperparameters are: ’num_feature=1’, ’max_depth= 10’, and ’booster=gblinear’.

Here, we applied the BO procedure for the OGPR, OSVR, and OEL models to get the optimal parameters maximizing the forecasting precision based on training data. The hyperparameter search ranges for each model, and the computed values of the hyperparameters of each model using the BO algorithm are summarized in Table 3. Specifically, the values of the hyperparameters are obtained by the MSE between the actual COVID-19 data and the predicted data during the training stage.

**Table 3:** Hyperparameters search range and Optimized Hyperparameters using the BO algorithm.

Model	Hyperparameter Search Range	Optimized Hyperparameters
SVRO	-Box constraint: 0.001-1000	-Box constraint: 1.7128
	-Kernel scale: 0.001-1000	-Kernel scale: 1
	-Epsilon: 0.18495-18495.1816	-Epsilon: 1.3156
	-Kernel function: Gaussian, Linear, Quadratic, Cubic	-Kernel function: Cubic
	-Standardize data: true, false	-Standardize data: true
GPRO	-Sigma: 0.0001-1441.9316	-Sigma: 1217.1288
	-Basis function: Constant, Zero, Linear	- Basis function: Linear
	-Kernel function: Exponential, Matern 5/2, Rational Quadratic, Squared Exponential	-Kernel function: Matern 5/2
	-Kernel scale: 0.498-498	-Kernel scale: 493.0376
	-Standardize: true, false	-Standardize: false
ELO	-Ensemble method: Bag, LSBoost	Ensemble method: LSBoost
	-Number of learners: 10-500	-Number of learners: 11
	-Learning rate: 0.001-1	-Learning rate: 0.98438
	-Minimum leaf size: 1-249	-Minimum leaf size: 2
	-Number of predictors to sample: 1-2	-Number of predictors to sample: 2

**Figure 8:**
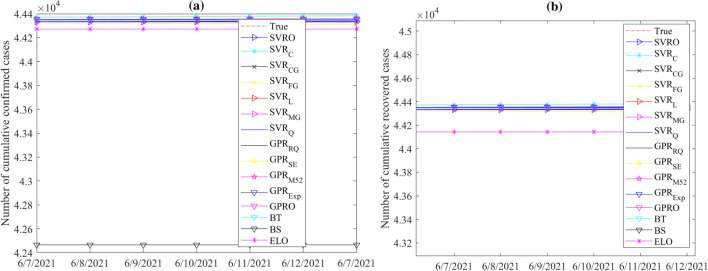
Records and forecasts of (**a**) confirmed and (**b**) recovered COVID-19 cases in India for testing period, using the fifteen machine learning methods.

**Figure 9:**
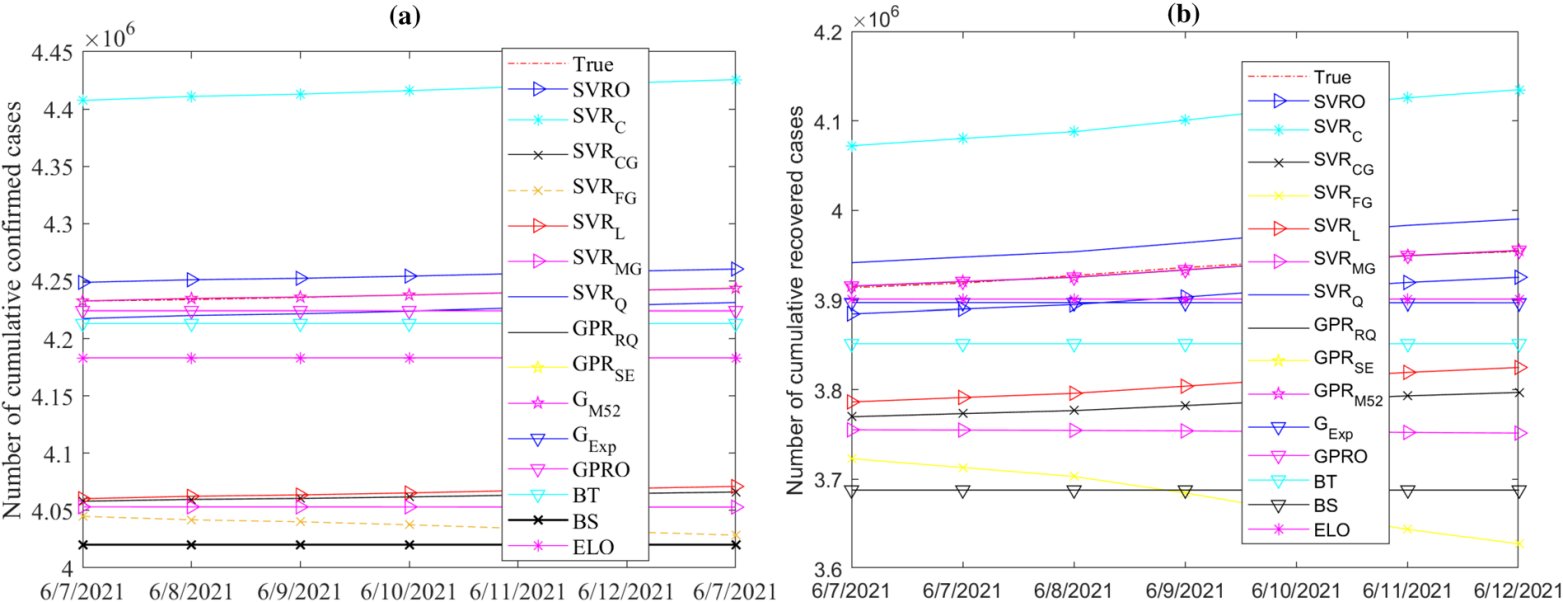
Records and forecasts of (**a**) confirmed and (**b**) recovered COVID-19 cases in Brazil for testing period, using the fifteen machine learning methods.

In this study, seventeen machine learning models (Table 2) are used to predict COVID-19 spread. We implemented these methods using Matlab R2021b. These models are first built based on training data and then used for forecasting confirmed and recovered COVID-19 cases for a forteen-day forecast horizon from May 30, 2021. We applied a 5-fold cross-validation technique in training the investigated models. Figures 8 and 9 display the recorded test set together with model forecasts of confirmed and recovered cases in India and Brazil, respectively. From Fig. 8, we observe that the forecasted values of the confirmed and recovered cases in India from the considered models are closer to the actual data, indicating good forecast performance. For the confirmed and recovered cases in Brazil, Fig. 9, shows broader bands around the actual cases, indicating wider variations among model predictions. In this scenario, models showed relatively better forecasts for India confirmed and recovered cases series.

Tables 4 and 5 quantifies the performances of each model in terms of RMSE, MAE, and MAPE, for COVID-19 data recorded in India and Brazil, respectively. In terms of all metrics calculated, the GPR models showed the best performance in RMSE and MAE. It could be attributed to their capacity to capture dynamics in time-series data.

**Table 4:** Th obtaine statistical criteria for confirmed and recovered COVID-19 cases forecasts in India.

Series	Model	RMSE	MAE	MAPE
Confirm India	SVRO	22337053.113	22334295.732	38.960
Confirm India	SVR$_{C}$	1012357.846	1008215.980	3.365
Confirm India	SVR$_{CG}$	1382637.913	1369677.347	4.967
Confirm India	SVR$_{FG}$	6967701.735	6507706.629	30.360
Confirm India	SVR$_{L}$	759414.577	759392.528	2.697
Confirm India	SVR$_{MG}$	2356188.304	2280681.541	8.581
Confirm India	SVR$_{Q}$	1024262.932	1019713.136	3.401
Confirm India	GPR$_{RQ}$	37398.517	32479.864	0.112
Confirm India	GPR$_{SE}$	36208.928	30442.130	0.105
Confirm India	GPR$_{M52}$	14350.001	12258.416	0.072
Confirm India	GPR$_{Exp}$	972208.519	905902.811	3.233
Confirm India	GPRO	111506.899	108951.780	0.374
Confirm India	BT	2005011.686	1974055.703	7.325
Confirm India	BS	2779609.635	2757363.556	10.538
Confirm India	ELO	1625388.219	1587044.713	5.806
Confirm India	RF	956649.730	853385.714	3.053
Confirm India	XGBoost	874210.275	759823.714	2.709
Recoved India	SVRO	21100097.634	21087507.583	11.320
Recoved India	SVR$_{C}$	1125965.648	1107063.877	3.919
Recoved India	SVR$_{CG}$	1771552.327	1718529.593	6.779
Recoved India	SVR$_{FG}$	10306345.167	9339278.915	59.969
Recoved India	SVR$_{L}$	754472.349	754424.474	2.877
Recoved India	SVR$_{MG}$	3670129.936	3373505.792	14.480
Recoved India	SVR$_{Q}$	1179022.579	1155346.231	4.080
Recoved India	GPR$_{RQ}$	167795.963	143454.775	0.527
Recoved India	GPR$_{SE}$	30214.921	23379.830	0.085
Recoved India	GPR$_{M52}$	54374.745	48482.147	0.178
Recoved India	GPR$_{Exp}$	1524148.063	1336405.700	5.208
Recoved India	GPRO	58832.766	46691.520	0.052
Recoved India	BT	3078226.027	2990707.504	11.681
Recoved India	BS	3467540.137	3390087.093	14.359
Recoved India	ELO	1871290.946	1723538.715	6.127
Recoved India	RF	1688270.003	1522862.929	5.977
Recoved India	XGBoost	1496590.266	1307148.929	5.088

**Table 5:** Th obtaine statistical criteria for confirmed and recovered COVID-19 cases forecasts in Brazil.

Series	Model	RMSE	MAE	MAPE
Confirm Brazil	SVRO	178495.629	176859.741	1.055
Confirm Brazil	SVR$_C$	859664.899	846897.020	4.749
Confirm Brazil	SVR$_{CG}$	856493.084	849454.839	5.279
Confirm Brazil	SVR$_{FG}$	2681791.796	2400829.100	17.153
Confirm Brazil	SVR$_L$	658423.358	657587.460	4.041
Confirm Brazil	SVR$_{MG}$	1201574.167	1157539.469	7.350
Confirm Brazil	SVR$_Q$	100175.382	92240.318	0.552
Confirm Brazil	GPR$_{RQ}$	22347.367	20542.504	0.122
Confirm Brazil	GPR$_{SE}$	36548.399	29617.766	0.175
Confirm Brazil	GPR$_{M52}$	25452.517	22951.994	0.136
Confirm Brazil	GPR$_{Exp}$	499989.497	426469.985	2.585
Confirm Brazil	GPRO	22821.043	21485.641	0.127
Confirm Brazil	BT	819117.717	776873.997	4.811
Confirm Brazil	BS	1414426.255	1390388.795	8.951
Confirm Brazil	ELO	1471998.737	1448916.717	3.363
Confirm Brazil	RF	503458.241	431334.642	2.615
Confirm Brazil	XGBoost	484044.544	408507.642	2.474
Recoved Brazil	SVRO	30627.182	30583.310	7.618
Recoved Brazil	SVR$_C$	167774.226	167591.124	4.806
Recoved Brazil	SVR$_{CG}$	151929.129	151847.006	5.351
Recoved Brazil	SVR$_{FG}$	259341.166	254940.374	18.745
Recoved Brazil	SVR$_L$	129890.658	129878.098	3.400
Recoved Brazil	SVR$_{MG}$	181760.427	181092.640	7.876
Recoved Brazil	SVR$_Q$	30362.953	30188.755	1.473
Recoved Brazil	GPR$_{RQ}$	1719.567	1558.378	0.241
Recoved Brazil	GPR$_{SE}$	1656.806	1525.704	0.247
Recoved Brazil	GPR$_{M52}$	1667.333	1508.890	0.188
Recoved Brazil	GPR$_{Exp}$	40549.196	37897.498	2.935
Recoved Brazil	GPRO	1667.329	1508.883	0.188
Recoved Brazil	BT	84573.683	83349.510	4.581
Recoved Brazil	BS	247302.171	246886.202	9.251
Recoved Brazil	ELO	36794.352	33885.945	3.489
Recoved Brazil	RF	26978.983	18038.854	2.452
Recoved Brazil	XGBoost	57643.591	36789.043	3.117

**Figure 10:**
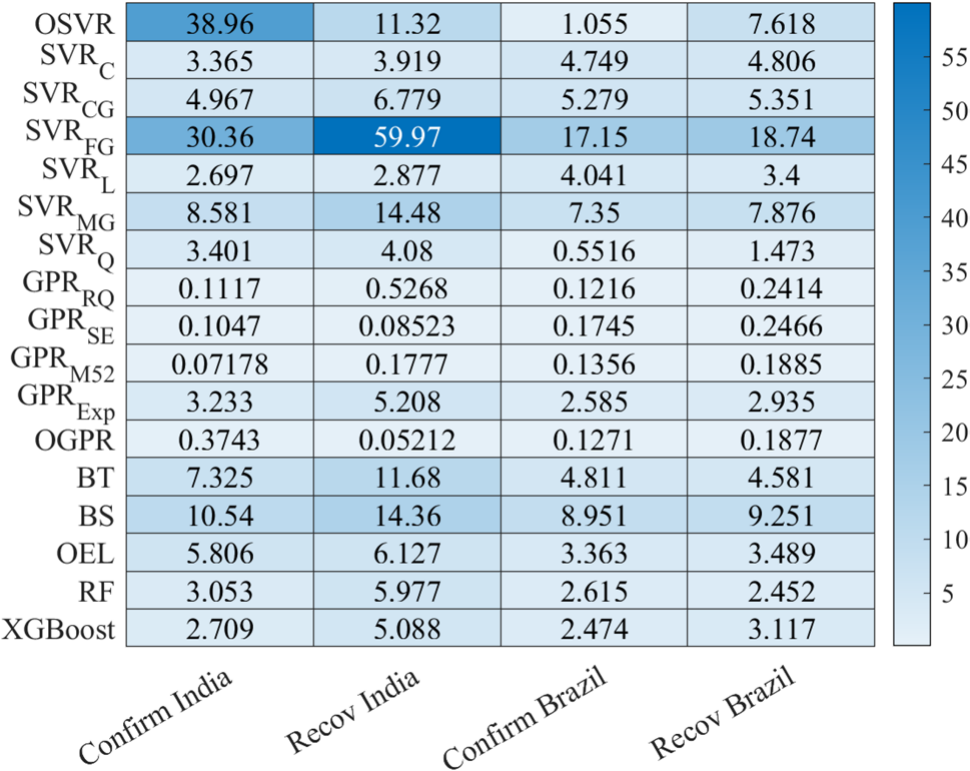
Heatmap of MAPE values obtained using the seventeen models.

Figure 10 displays the heatmap of the MAPE values achieved by the investigated model for the confirmed and recovered COVID-19 data from Indian and Brazil. We observe that GPR models achieved the best forecasting performance with the lowest MAPE values. This could be attributed to the extended capacity of the GPR models to learn dynamics in COVID-19 time-series data. Furthermore, this study shows the capability of machine learning models to forecast the future trends of COVID-19.

**Figure 11:**
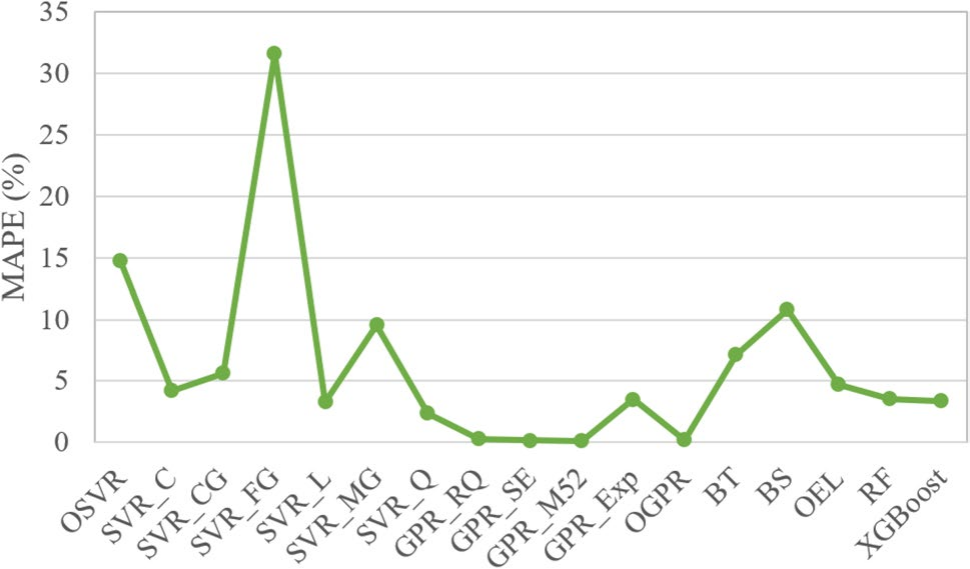
Averaged MAPE per model.

Figure 10 indicates that there is not a unique approach that is uniformly superior to others. For instance, the GPR$_{M2.5}$ achieved the best results for confirmed cases in India and recovered cases in Brazil, and OGPR obtained the best accuracy for recovered cases in India and confirmed cases in Brazil. Thus, averaged MAPE values per model are provided for comparison to find the best model. Figure 11 depicts the averaged MAPE per model. The lowest average MAPE value characterizes the best model. The average MAPE of the OGPR mode is 0.185%. For SVR models, the best prediction is obtained by using the SVR$_{Q}$ with an average MAPE of 2.376%. The average MAPE of XGBoost, RF, and OEL models are 3.347%, 3.524%, and 4.696%, respectively. Importantly, results in Fig. 11 highlight that a satisfactory forecasting COVID-19 spread can be obtained using shallow and simple machine learning approaches. In addition, it is easy to see that the GPR with optimized parameters using Bayesian optimization exhibited superior performance.

### Dynamic model

**Figure 12:**
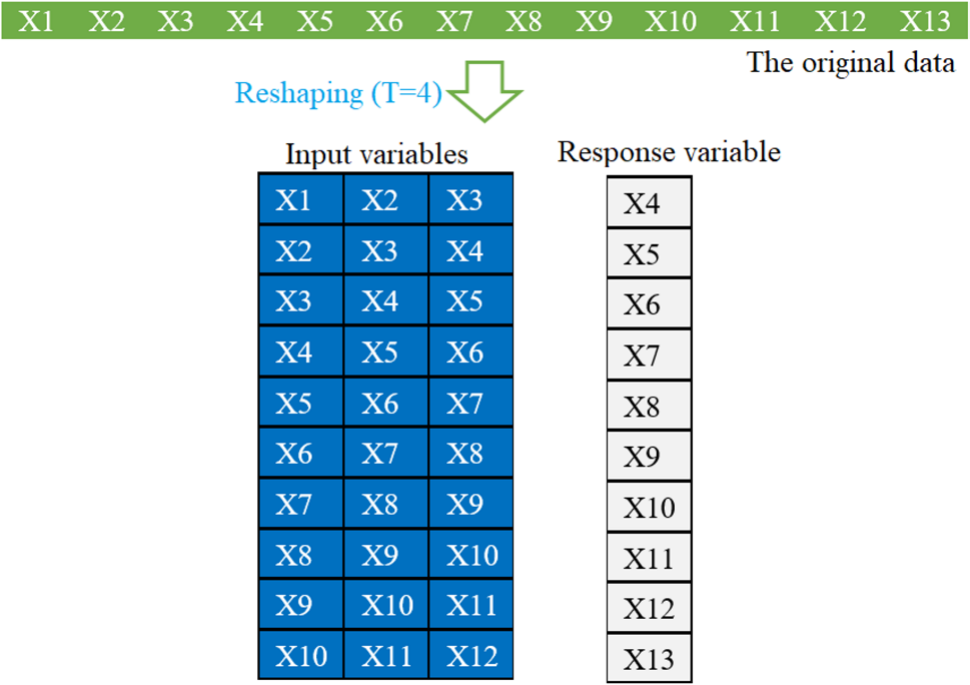
Process of dataset preparation for dynamic models.

Note that the abovementioned results are based on static models that ignore information from the past data. In this section, we investigate the performance of the machine learning models when incorporating information from the past data in model construction. In other words, to capture the dynamic and evolving nature of the COVID-19 time series, we introduce lagged data when building the prediction models. Here, we apply a dynamic fifteen models on India and Brazil dataset by considering the past days’ interval. As in the static model, we used the last fourteen days from May 30, 2021, to June 12, 2021, for the testing. Figure 12 shows how the past data can be incorporated into the input data; here is an example of adding the information from the past three days in the input data. In this case study, we evaluate the impact of introducing past information (i.e., one day, two days, three days, four days, five days, six days, and seven days) on the prediction performance of the investigated models.

**Figure 13:**
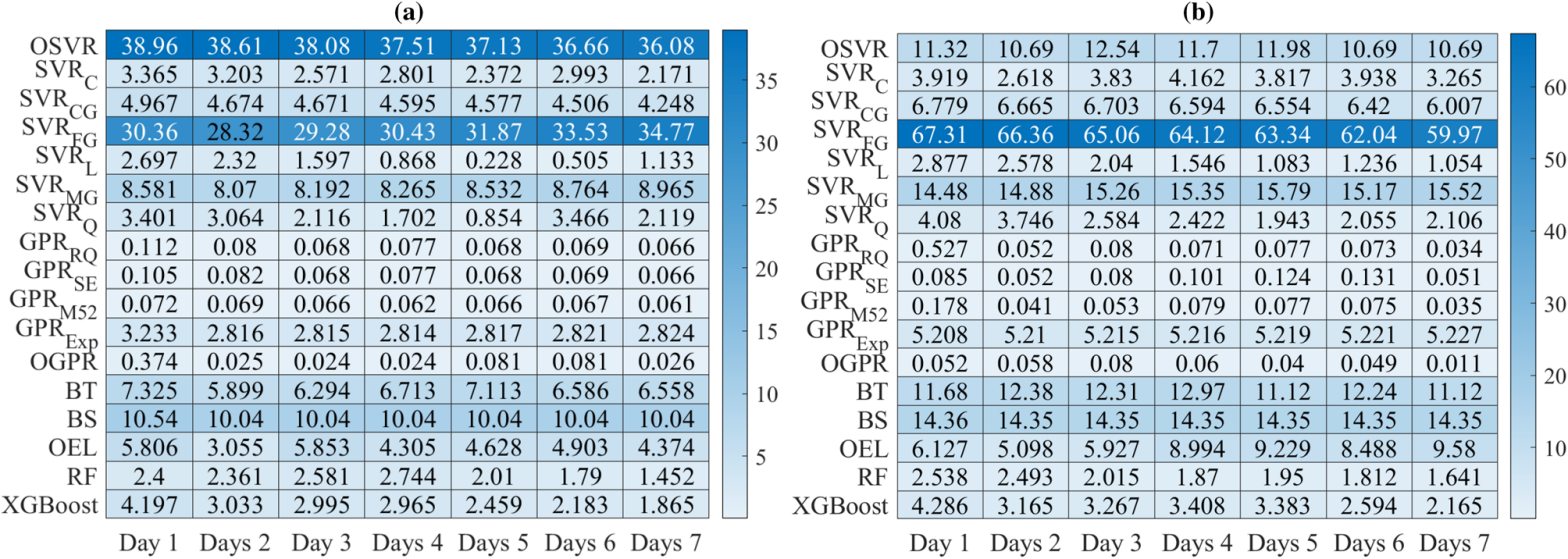
HeatMap of MAPE values by methods for (**a**) Confirmed and (**b**) recovered COVID-19 times series in India.

**Figure 14:**
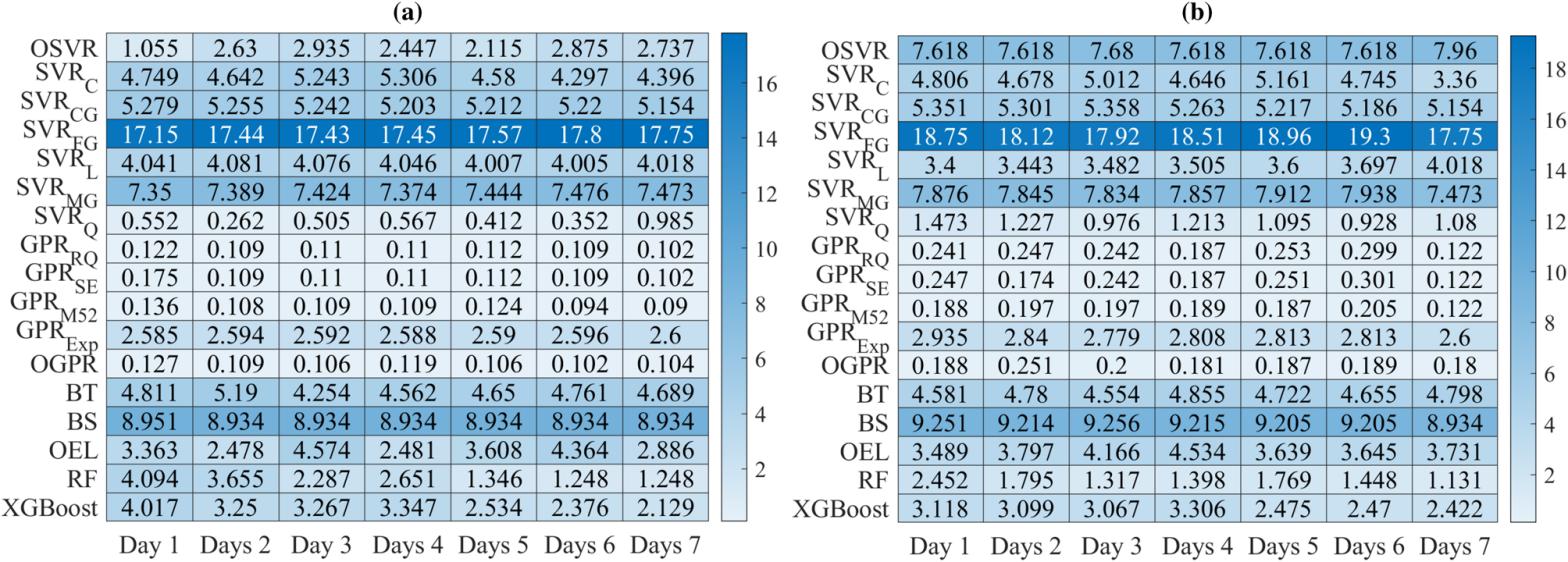
HeatMap of MAPE values by methods for (**a**) Confirmed and (**b**) recovered COVID-19 times series in Brazil.

**Figure 15:**
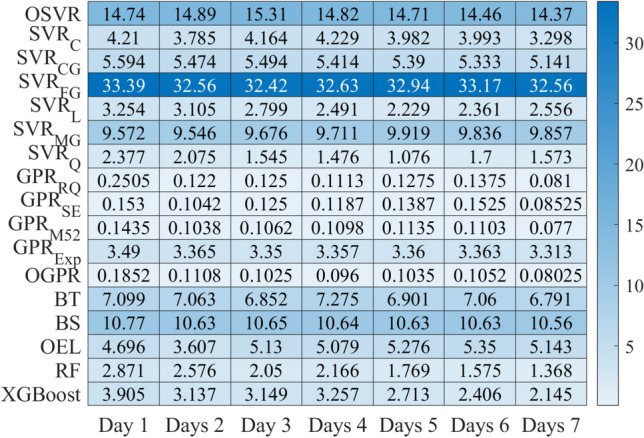
Averaged MAPE values per model.

Figures 13 and 14 show the MAPE performances values of each model when applied to forecast COVID-19 data recorded in India and Brazil from 1 to 7 days, respectively. It can be seen that incorporating information from past data improves the forecasting performance compared to the static model, and the MAPE values decrease, which means that prediction performance has been improved. Prediction results in Figs. 13 and 14 confirm that incorporating information from past data improves forecasting quality compared to the static models. Figure 15 illustrates the averaged MAPE values per model and shows that GPR models exhibited the highest forecasting accuracy among all other models by reaching the lowest MAPE values. Also, we can see that GPR$_{M52}$ and GPRO reached relatively similar performance and outperformed the other models. In short, this demonstrates the ability of GPR models to learn dynamics in COVID-19 time-series data.

**Figure 16:**
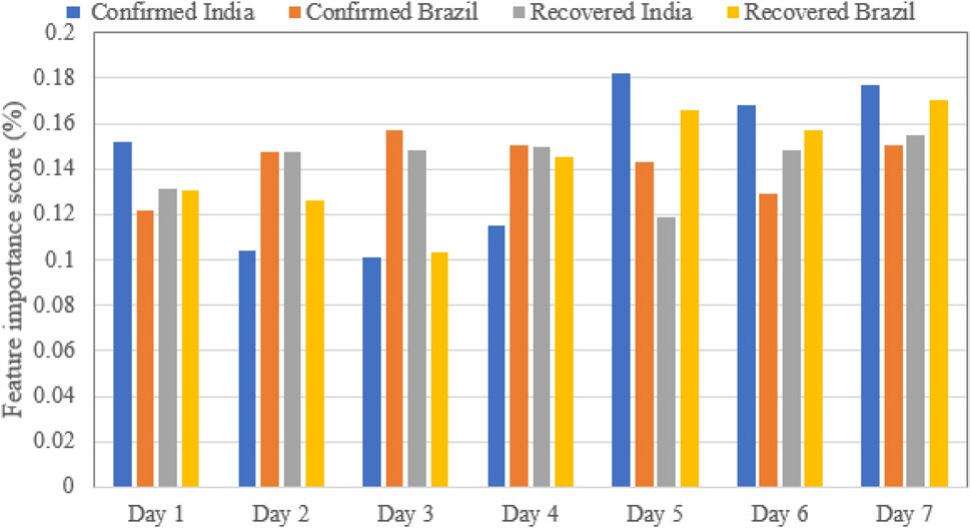
Feature importance identification based on RF by time-series.

As shown above, considering information from past data is constructing prediction models leads to improved prediction performance. Now, it is crucial to identify the most important features for prediction. Indeed, this will enables removing unnecessary features and constructing parsimonious models. Here, Random forest (RF) will be applied to evaluate the impact of each variable on the prediction of COVID19 spread. It used the Recursive feature elimination algorithm to identify the weights of the features and rank the features according to the importance weights^78,79^. Figure 16 shows the variable importance score when applying RF to COVID-19 India and Brazil dataset. Importantly, the seven past days (features) are relatively impacting the prediction at a similar level. Moreover, from Fig. 16, reduced dynamic models that incorporate information from the past six days will be able to sufficiently capture the future tend of COVID-19 time-series data.

**Figure 17:**
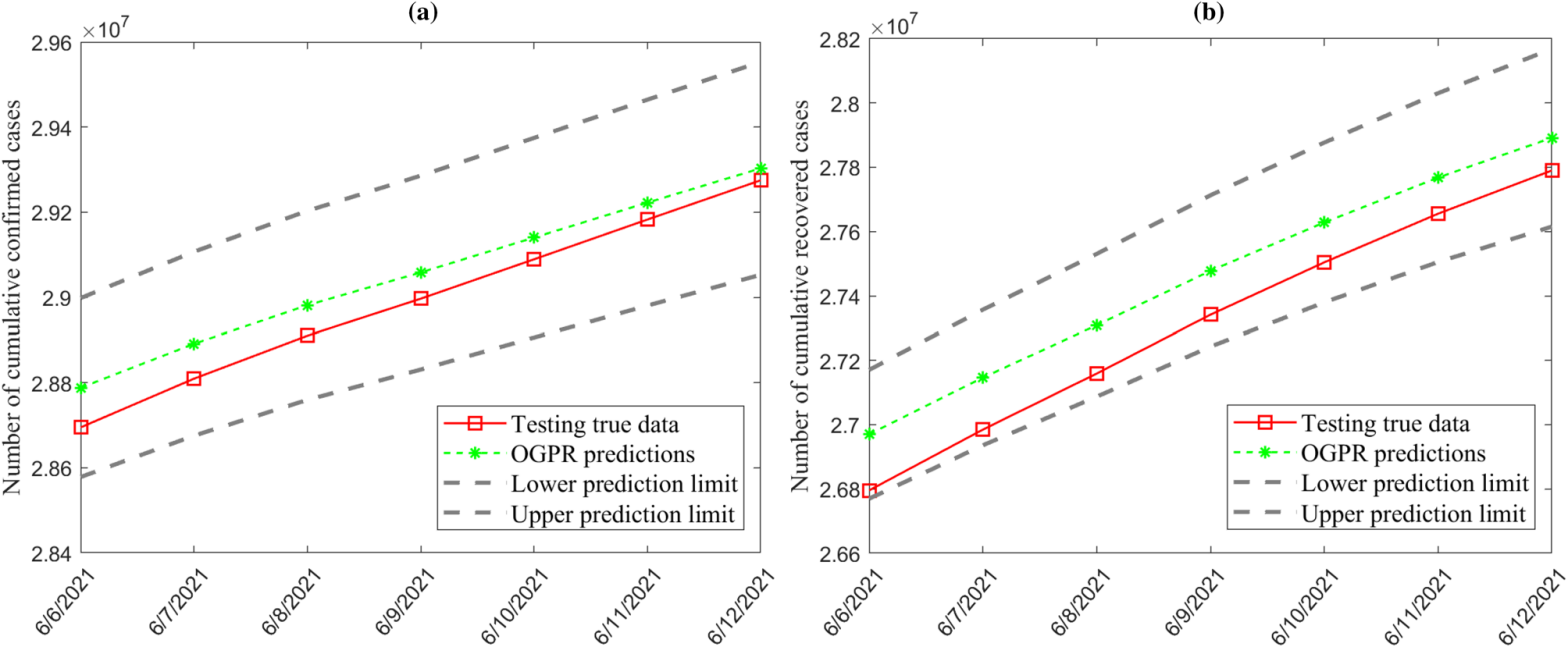
One-step-ahead prediction boundaries for (**a**) confirmed cases and (**b**) recovered cases in India with the GPR model.

Figure 17 displays the one-step-ahead prediction results of the dynamic OGPR model of the confirmed cases and recovered cases in India based on testing data. It can be seen that the OGPR predicted values are in agreement with the recorded COVID-19 values. In addition, the predicted values are very close to the observed values, and both of them are inside the 95% confidence intervals. It should be noted that this information cannot be obtained using ensemble models and SVR models, which makes dynamic SVR models very helpful. Results are very promising and confirm that the predicted COVID-19 cases closely follow the recorded COVID-19 trends. Also, these results reveal the importance of optimizing the GPR model with BO and incorporating information from past data to achieve the best prediction performance.

In summary, in this study, both static and dynamic machine learning models have been investigated for predicting COVID-19 spread in two highly impacted countries, India and Brazil. It was concluded that both dynamic and static model prediction models considered in this work could predict COVID-19 spread with a satisfactory degree of accuracy. Importantly, results revealed that the dynamic models that incorporate past data information result in lower prediction errors than the static models. Specifically, the dynamic optimized GPR model outperformed all the other considered static and dynamic prediction models in predicting COVID-19 spread in India and Brazil.

As discussed above, various methods have been developed to improve the forecasting of COVID-19 spread using machine learning and time-series models^4,28,30–34^. Table 6 summarized different studies on COVID-19 time series forecasting. Table 6 compares the achieved average MAPE of the proposed dynamic OGPR model with those of the state-of-the-art methods. It should be noted that the average MAPE of the SOTA methods listed in Table 6 are computed from the provided MAPE values in the original papers. Note that as the used COVID-19 training and testing data are not the same, it is not easy to compare the performance of the proposed approach with the SOTA methods. However, the summary in Table 6 is helpful to get a big picture about the forecasting performance of the existing methods when applied to small-sized datasets. Table 6 shows that time-series models in^30,32,34^, such as ARIMA, SES, HW, BATS, PAR, and polynomial, obtained lower accuracy in range 13.3%-47.415%. On the other hand, an ARIMA model in^31^ showed moderately high prediction with a MAPE of 5.59%. These results could be due to different factors, including parameters setting and the size of data used for model training. The amount of data employed in these studies is relatively small. From Table 6, it is interesting to see in^28^ that a linear regression model reached high prediction performance (i.e., MAPE of 0.2228%) since the COVID-19 outbreak is often considered as having exponential dynamics. The shallow machine learning methods employed in^28^ (i.e., RF, MLP, and SVM) showed good performance by obtaining an averaged MAPE in the range of 0.1162-1.0042. However, it can be seen that SVM and MLP obtained relatively low prediction performance with MAPE values 23.5 and 17, respectively. As data-driven approaches, the quality and amount of data are essential to construct a good predictive model. In addition, tuning the hyperparameters in training is crucial to obtain an efficient model that captures the most variability in training data and can predict future trends of COVID-19 spread. Results in^4,37,38^ show that RNN-based models, including LSTM, GRU, and GAN-GRU, LSTM-CNN, have sufficient ability in solving this limited size univariate time series forecasting problem with high efficiency and satisfying precision (i.e., MAPE values within 0.6203–5.254%). However, it is worth noting that deep learning methods, such as LSTM and GRU, are designed to capture long-term dependencies in time series data; they could provide enhanced prediction when implemented using a large amount of data. Overall, the proposed OGPR approach achieved high forecasting accuracy with an average MAPE of 0.1025%. Overall, the proposed OGPR approach achieved high forecasting accuracy with an average MAPE of 0.1025%. It could be attributed to different factors: i) the GPR as a distribution-free learning model can be applied to handle not necessarily normally distributed data, ii) it has good capacity to address difficult nonlinear regression problems via kernel trick, iii) it considers dynamic information by incorporating lagged data as input, and iv) provide better prediction when the hyperparameters are optimized using Bayesian optimization algorithm. Thus, it can be deduced that the proposed approach presents a promising system to forecast COVID-19 spread.

**Table 6:** Summary of different studies on COVID-19 spread prediction.

Refs	Country	Model	Average MAPE (%)
Ceylan^31^	Italy, Spain, and France	ARIMA	5.59%
Ballı Serkan^28^	Germany and USA	Random forest	1.0042
		Linear Regression	0.2228
		MLP	0.5153
		SVM	0.1162
Nasution et al.^30^	Jakarta	ARIMA	20.51
		SES	20.435
		HW	47.415
		BATS	33.945
		Prophet	42.27
		PAR	18.435
Istaiteh et al.^34^	China, Eritrea	ARIMA	14.14
		ANN	3.23
		LSTM	4.14
		CNN	3.13
Shaikh et al.^32^	India	Linear regression	27.9
		Polynomial with 2 degrees	13.3
Acosta et al.^33^	Brazil, Chile, Colombia, Mexico, Peru and the United States	SVM	23.5
		MLP	17
Dairi et al.^4^	Brazil, France, India, Mexico, Russia, Saudi Arabia, and the US	RBM	18.452
		CNN	20.763
		LSTM	20.394
		GAN-DNN	11.105
		GAN-GRU	5.254
		LSTM-CNN	3.718
Omran et al.^38^	Egypt, Saudi Arabia, Kuwait	a single-layer GRU	3.0419
		a single-layer LSTM	0.6203
Kafieh et al.^37^	Nine countries, including China, Spain, Italy, and the US	M-LSTM	0.509
**Proposed**	India and Brazil	**OGPR**	**0.1025**

## Conclusion

Accurate forecasting of COVID-19 spread is a key factor in slowing down this pandemic’s transmission by providing relevant information to help hospital managers make decisions and appropriately manage the available resources and staff. This work aimed to develop an effective data-driven approach to predict the number of COVID-19 confirmed and recovered cases in India and Brazil, ranked as the second and third countries with the highest number of confirmed cases behind the United States. This paper introduces a dynamic GPR model with optimized hyperparameters via Bayesian optimization into COVID-19 spread forecasting. Other promising prediction models, such as SVM, GPR, Boosted trees, Bagged trees, RF, and XGBoost, were also considered based on the same data. Here, the considered machine learning models are distribution-free learning methods that can be employed with no prior assumption on the data distribution. The SVR and GPR are within kernel-based prediction methods, while Boosted, Bagged trees, RF, and XGBoost are within ensemble learning methods. The SVR modeling is based on solving a nonlinear optimization problem; on the other hand, the GPR model uses Bayesian learning. This study investigates two types of prediction models, static and dynamic models, to improve COVID-19 forecasting accuracy. The static model ignores the information from past data, whereas dynamic models consider information from lagged data in forecasting COVID-19 spread. The results showed that the dynamic GPR models outperformed the other static and dynamic models in all cases. In short, the forecasting result shows that the optimizable GPR model is the winner model that achieved the best performance among the other models in terms of RMSE, MAE, and MAPE. In addition, the dynamic OGPR-based prediction models enable generating predictions with confidence intervals. This information is relevant and enables evaluating the reliability of the COVID-19 spread predictions and for making better use of the forecasted data. The overall prediction accuracy of the suggested dynamic OGPR model has been satisfying.

Despite the satisfactory COVID-19 spread forecasting results using the dynamic machine learning models, there is still plenty of room for improvement. At first, the suggested OGPR approach needs to be employed in more countries to confirm its superior performance. Moreover, accurate modeling of temporal and spatial dynamics of the COVID-19 spread is necessary to understand its spread in space-time for improved risk management. As the developed methods ignore the spatial spatio-temporal correlation in the COVID-19 spread, we plan to develop a more flexible forecasting approach that considers spatio-temporal correlations and mobility information in constructing machine learning methods to improve the forecasting quality of COVID-19 spread. Another direction of improvement is to incorporate external factors that affect the number of COVID-19 cases, such as the number of administered vaccines, the country’s population, medical resource availability, and government policies.
